# Recruitment of Both the Mirror and the Mentalizing Networks When Observing Social Interactions Depicted by Point-Lights: A Neuroimaging Study

**DOI:** 10.1371/journal.pone.0015749

**Published:** 2011-01-10

**Authors:** Laurie Centelles, Christine Assaiante, Bruno Nazarian, Jean-Luc Anton, Christina Schmitz

**Affiliations:** 1 Laboratoire Neurosciences Intégratives & Adaptatives - Université de Provence & Centre National de la Recherche Scientifique, Marseille, France; 2 Laboratoire Motricité Adaptation et Cognition - Centre National de la Recherche Scientifique, Bordeaux, France; 3 Centre d'IRM Fonctionnelle Cérébrale - Centre Hospitalier Universitaire de la Timone, Marseille, France; Cuban Neuroscience Center, Cuba

## Abstract

**Background:**

Understanding social interactions requires the ability to accurately interpret conspecifics' actions, sometimes only on the basis of subtle body language analysis. Here we address an important issue that has not yet received much attention in social neuroscience, that of an interaction between two agents. We attempted to isolate brain responses to two individuals interacting compared to two individuals acting independently.

**Methodology/Principal Findings:**

We used minimalistic point-light displays to depict the characters, as they provide the most straightforward way to isolate mechanisms used to extract information from motion per se without any interference with other visual information. Functional magnetic resonance imaging (fMRI) method was used to determine which brain regions were recruited during the observation of two interacting agents, mimicking everyday social scenes. While the mirror and mentalizing networks are rarely concurrently active, we found that both of them might be needed to catch the social intentions carried by whole-body motion.

**Conclusions/Significance:**

These findings shed light on how motor cognition contributes to social cognition when social information is embedded in whole-body motion only. Finally, the approach described here provides a valuable and original tool for investigating the brain networks responsible for social understanding, in particular in psychiatric disorders.

## Introduction

Non-verbal communication participates considerably to our understanding of social situations. Gaze direction, facial expressions, gesture and posture, all provide relevant cues that are part of body language [1]. Among these components, it is striking how much information whole-body movements can carry in terms of their social content: subtle differences in the kinematics of an index finger raised up can result in completely different interpretations, from showing an object to kicking someone out of a room. The present study aims at delineating the brain regions participating in the understanding of social intentions carried by body language only. Agent's social intentions were defined as intentions to act on conspecifics, who, unlike inanimate targets of action, can act back [2].

In the seventies, Johansson defined a new and original method to study human movement features, using point-lights [3]. As such, the term “biological motion” refers to movements of humans (or animals) displayed by point-lights solely. This enables to extract information from motion *per se* without any interference from other visual cues. Human motion provides important information about the meaning of actions [4], the gender and identity of the characters [5]–[8], and their emotional states [9]–[12].

Perception of whole-body point-light displays specifically involves the posterior part of the superior temporal sulcus (pSTS) [13]–[15]. Interestingly, it also triggers activities in the action observation/execution matching network which is activated both when an action is observed and performed [16]. This network consists of the premotor cortices (PM) [17], [18], the inferior frontal gyrus (IFG) [19], and parietal regions [20], [21]. As these regions contain mirror neurons [16], [22], this network is referred to as the mirror system. This system might be the neural substrate of the motor simulation theory (i.e., the motor resonance) [23], [24]. According to this theory, people understand the goal of other people's actions by simulating these actions with their own motor programs [23], [25]–[28]. When it comes to social interactions, the observer needs to understand the intentions carried by two persons, challenging the ability to simultaneously match both actions onto his own motor repertoire [2]. So far, whether motor simulation contributes to understanding the social intentions generated by two interacting persons has still not been clearly demonstrated [2], [29], [30].

The ability to process any information which leads to the perception of a social feature is subserved by a set of brain regions also defined as the social brain [31]. Among these structures, the medial prefrontal cortex (MPFC), the pSTS and the temporo-parietal junction (TPJ), the anterior superior temporal sulci (aSTS) and the amygdala play a major role [32]–[35]. Some of them are thought to be devoted to mentalizing processes, but the findings are heterogeneous and implicate an anatomically broad set of regions [36].

The major objective of the present study was to clarify the contribution of several key brain areas to the understanding of everyday social interactions perceived through whole-body human motion. Specifically, we hypothesized that the activity of the mirror system would be enhanced by the presence of a social interaction, because it requires understanding the actions and intentions of two agents at the same time, therefore presenting with a greater complexity of action understanding. Functional magnetic resonance imaging (fMRI) was carried out while subjects were observing point-light displays in which two agents performed together everyday social scenes or moved independently without interacting. Two situations were compared in which two agents were either interacting together (the social interaction condition, SI) or moving separately without expressing any social intention (the no social interaction condition, NSI).

## Materials and Methods

### Subjects

Fourteen healthy adult subjects (six females and eight males, mean age 29.4±7.4 years) took part in this study. All the subjects had normal or corrected-to-normal visual acuity and no significant history of medical, psychiatric or neurological illness. All participants gave their written informed consent prior to participating in the study, which was approved by the local ethics committee (Comité de Protection des Personnes Sud Méditerranée 1).

### Stimuli

Three-second silent point-light displays were created by videotaping two different professional actors (2 females). The displacement of twenty markers (15 mm in diameter) taped onto the actors' bodies was recorded with the SMART automatic motion analyzer (BTS) at 120 Hz. This resulted in each actor being depicted by twenty white dots (top of the head, neck, shoulders, elbows, wrists, hands, thoracic and sacral vertebra, hips, knees, ankles, little toes), moving against a black background.

**Figure 1:**
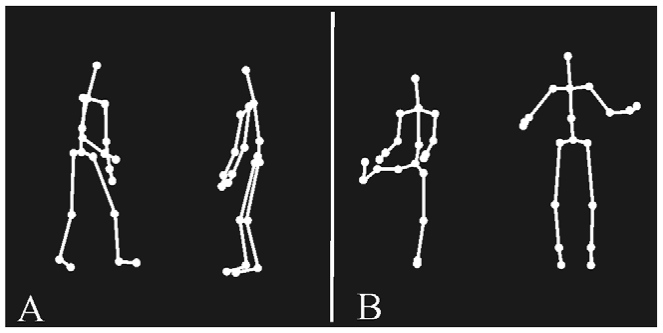
Point-light displays showing social interactions (A) and no social interactions (B). The example of SI depicts an actor showing something on the ground to another actor, who responds by coming closer. The example of NSI depicts one actor raising a leg and the second one jumping. In this illustration, in order to help to distinguish the human form, the dots have been linked by full lines.

The two actors were either interacting together (SI) (Fig. 1A, Videos S1, S2, S3) or moving side by side without interacting (NSI) (Fig. 1B, Videos S4, S5, S6). SI consisted in a dynamic sequence in which one of the actor performed an action which triggered a reaction from the other actor, resulting in a meaningful social scene. The aim was to portray ecological, lifelike everyday social scenes. A large range of SI displays were therefore used to cover social scenes under the heading of social norms (conventional gestures and courteous attitudes), emotional situations (carrying positive or negative valences) and scenes from games (sports, dance, etc.). Note that objects (such as a balloon, sword and chair) were sometimes used but not visible. Twenty social screenplays as defined above were played by the actors. Each original screenplay was played twice since each of the actor initiated the social interaction in turn. In the second condition (NSI), the two same actors performed non goal-directed movements without interacting. These movements included raising an arm or a leg, rotating the trunk, bending forward or sideways, stepping forward or backward, jumping, and other movements with no social connotations. The two actors were filmed separately before being pasted side by side in the NSI scene in order to prevent any synchronization of movements which might have induced the perception of social exchanges between the two characters [37]. Stimuli used for SI and NSI conditions were matched for overall motion speed and actors' types of movements as closely as possible.

We also used two different viewpoints when capturing the original movies in order to vary the observer's viewpoint. In the SI scenes, the two agents were either face-to-face or the one was turned sideways with respect to the other. In the NSI scenes, the two agents were either side-by-side or the one was turned sideways with respect to the other. They were never presented moving face-to-face in the NSI scenes in order to rule out the possibility that their actions might be mistakenly perceived as social interactions.

Prior to the fMRI experiment, a behavioral study (pre-fMRI study) was performed to test the relevance of the stimuli described above by evaluating the recognition accuracy. Subjects were asked to classify the stimuli as NSI or SI. Any ambiguous stimuli were eliminated on the basis of the error rate, i.e., more than 20% made by the group. A resulting set of 112 stimuli was used for the fMRI experiment. The 56 SI displays and the 56 NSI displays were equally divided into 4 runs. An additional set of 28 stimuli was used during the practice session, which was carried out prior to scanning the subjects.

### fMRI task and procedure

**Figure 2:**
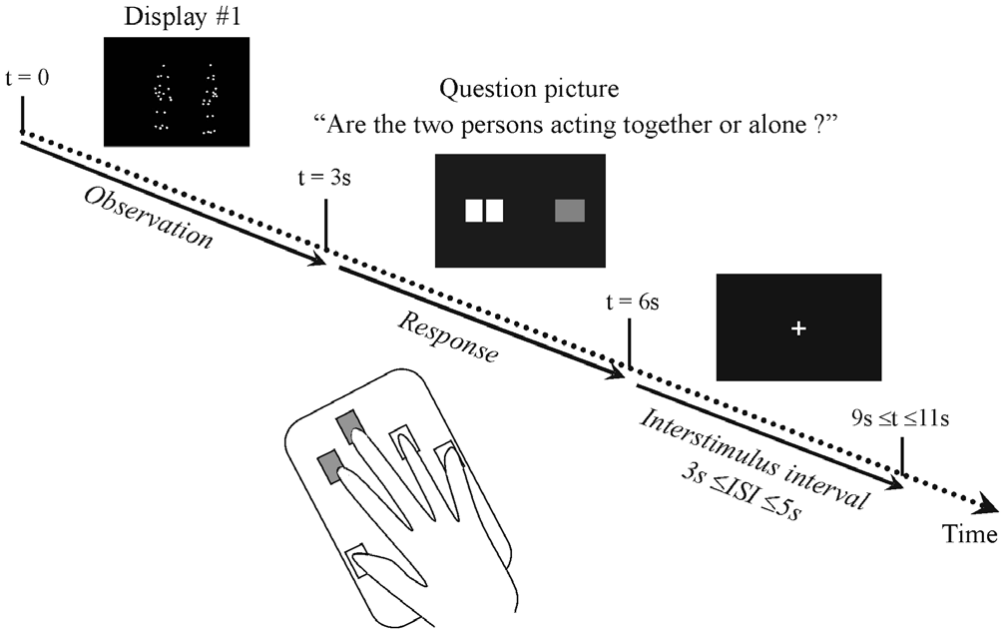
Sequence of trial presentation across time. The training sequence contained a plain green rectangle (shown in grey in the figure) and a red rectangle (in white here) divided into two parts. The plain green rectangle symbolized the two persons acting together; and the red rectangle divided in two parts stood for the two agents moving independently.

The experiment had an event-related design composed of the two conditions SI and NSI. A scheme of the procedure is given in Fig. 2. Following a fixation cross, each scene was shown for 3 s in random order. Subjects were asked to watch the displays carefully and to categorize them. An instruction sequence, consisting of a plain green rectangle and a red rectangle divided into two parts, appeared for 3 s thereafter. The plain green rectangle symbolized the two persons acting together, and the red rectangle divided into two parts stood for the two persons moving independently. Each rectangle could appear on the right or left side of the screen, at random in order to avoid anticipatory responses and to maintain the attention of the subject constant. The subject's right hand was placed above a response pad with the index finger placed above the left button and the middle finger above the right button. The participants had to answer the question “Are the two persons acting together or separately?” by pressing the button on the side where the rectangle they chose was presented. For instance, if they thought the two persons were acting together and the green rectangle was presented on the left side of the screen, they had to press the left button. During the inter stimulus interval (which lasted between 3 and 5 s at random), the fixation cross appeared again.

The stimuli were back projected onto a frosted screen positioned at the back end of the MRI tunnel and viewed by the subjects through a mirror. Stimuli were presented and responses recorded using a software program based on the LabVIEW 7.1 development system. Before scanning, participants were first instructed and trained to classify the two types of displays. A debriefing session was run immediately after each experiment to ensure that the participants had encountered no difficulties while performing the task in the scanner.

### fMRI data acquisition

Images were acquired on a 3-T MEDSPEC 30/80 AVANCE whole-body imager (Bruker, Ettlingen, Germany) equipped with a circular polarized head coil. Participants were lying comfortably in the supine position in the MR scanner. An ergonomic MR compatible response pad was placed under the subject's right hand. Headphones were provided to dampen the scanner noise and to be able to communicate with the subject. Anatomical MRI data were acquired using high-resolution structural T1-weighted image (inversion–recovery sequence, resolution 1×0.898×1.422 mm) in the sagittal plane, covering the whole brain. For functional imaging, a T2*-weighted echo planar sequence covering the whole brain with 32 interleaved 3-mm-thick/1 mm-gap axial slices (repetition time  = 2133.3 ms, echo time  = 30.0 ms, flip angle  = 79.5°, FOV  = 192 mm, 64×64 matrix of 3×3×4 mm voxels) was used. Four runs, each including 14 different SI and 14 different NSI, were conducted with each subject. At each run, lasting 4 mn50 s, 137 functional volumes were acquired. The whole experiment lasted for about forty minutes.

Data were processed and analyzed using SPM2 (Wellcome Department of Imaging Neuroscience, Institute of Neurology, London; http://www.fil.ion.ucl.ac.uk/spm). The first six functional volumes acquired in each runwere discarded to ensure that longitudinal relaxation time equilibration was achieved. The remaining 131 images were corrected for differences in slice acquisition time. A slice acquired half-way was chosen as reference in order to correct for temporal differences between the first and last slices. All volumes were realigned to the first volume to correct for head movements between scans. The functional images were then co-registered to each individual anatomical T1-weighted image and spatially normalized to the Montreal Neurological Institute (MNI) standard space. Data were then spatially smoothed using an 8 mm full-width at half-maximum isotropic Gaussian kernel to accommodate for inter-subject differences in anatomy.

### Statistical analysis

Given the high success rates obtained in recognizing both SI and NSI (more than 95% of the responses were correct, see Results section) and the lack of significant differences, we pooled accurate and inaccurate answers in our statistical model. The statistical analysis of the pre-processed BOLD signals was performed using a generalized linear model (GLM) approach. Three regressors were modeled using a 3 s box-car waveform convolved with the canonical hemodynamic response function (HRF). Two regressors (SI and NSI conditions) were considered as events of interest while the regressor modeling the subject's answer was considered as an event of no interest. Since we were interested in the social components of human motion, and as the NSI condition was considered as the control condition, only statistical images corresponding to the (SI -NSI) contrast were computed.

To account for inter-subject variability in the group analysis, the contrast images obtained at level 1 were included in a second level t-test, to create an SPM map. A one-sample t-test was used. All the fMRI statistics and *p* values were based on group random-effect analyses. The false-discovery-rate (FDR) [38] threshold of *p*<0.05 was adopted to deal with the problem of multiple comparisons by automatically defining a statistical significance threshold ensuring that the average rate of false positives among the voxels activated would be less than *p*. Activated brain regions were defined as clusters consisting of more than 10 contiguous voxels. The anatomical localization of the activations was determined using the atlas of Duvernoy [39], according to the major sulci and gyri distinguishable on a representative normalized anatomical MRI of one subject.

### Behavioral data

Subjects' performances (whether they opted accurately between SI and NSI) were rated in terms of the percentage of accurate responses obtained in each condition. Comparisons were performed using the non-parametric Wilcoxon signed-rank test. Since performances could be in the 0 to 100 range, we used a z transform to convert the values into an unbiased Gaussian distribution.

## Results

### Behavioral performances

The average percentages of correct answers were: SI  = 96.7% (SD  = 3.8) and NSI  = 99.0 (SD  = 1.3). Statistical analyses did not show any difference between the two conditions (W = 43 with *p* = 0.092). Since they were not significantly different, the two conditions could be taken to be equally easy.

### Brain activations: Social interaction (SI) versus No social interaction (NSI)

**Figure 3:**
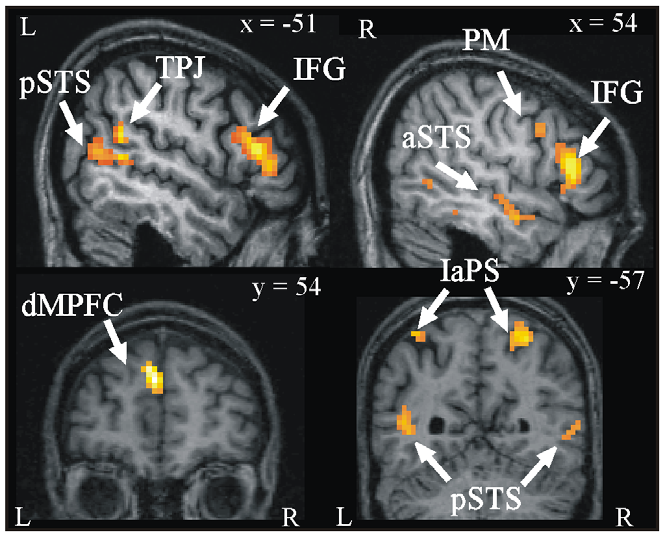
fMRI results from random-effects analyses: *social interaction* > *non social interaction*. Significant activations (*p<0.05*, corrected) in the whole-brain random effects analysis during the observation of social interaction displays (SI) versus non social interaction displays (NSI). Group activations are projected onto the normalized anatomical brain of one of the participants. pSTS  =  superior temporal sulcus (posterior part), TPJ  =  temporo-parietal junction, IFG  =  inferior frontal gyrus, PM  =  premotor cortex, aSTS  =  superior temporal sulcus (anterior part), dMPFC  =  medial prefrontal cortex (dorsal part), IaPS =  intraparietal sulcus, R/L =  right and left hemispheres.

The main effect of watching animations depicting social interactions (SI) in contrast to animations without social interactions (NSI) elicited significant patterns of activation, as shown in Fig.3 and listed in Table 1.

**Table 1:** Brain areas showing increased activity in response to the social interaction condition.

Side	Brain regions	MNI coordinates			Brodmann area	*t* value	Cluster size
		X	Y	Z			
L	Medial prefrontal cortex, dorsal part	−3	54	27	BA9/10	8.91	73
R	Lateral orbitofrontal gyrus	24	15	−18	BA11/47	7.29	27
L	Lateral orbitofrontal gyrus	−39	24	−15	BA11/47	4.96	40
L	Inferior frontal gyrus, *pars triangularis*	−51	30	12	BA45/46	7.14	189
L	Inferior frontal gyrus, *pars opercularis*	−45	3	18	BA44/45	5.57	
R	Inferior frontal gyrus, *pars triangularis*	54	27	9	BA45/46	6.74	67
R	Medial supplementary motor area	3	12	54	BA6	5.71	59
L	Medial supplementary motor area	−9	21	57	BA6	4.17	
L	Premotor cortex, dorsal part	−30	0	57	BA6	5.42	14
L	Premotor cortex, ventral part	−45	−3	54	BA6	4.62	15
R	Premotor cortex, ventral part	45	12	42	BA6	4.67	13
R	Superior parietal gyrus	24	−60	63	BA7	6.43	88
R	Intraparietal sulcus	24	−51	57	BA7/40	4.72	
L	Intraparietal sulcus	−42	−48	54	BA7/40	6.43	30
R	Superior temporal sulcus, posterior part	42	−72	18	BA39	5.42	42
L	Temporo-parietal junction	−51	−51	21	BA39	5.81	134
L	Superior temporal sulcus, posterior part	−48	−63	12	BA21/22	5.12	
R	Superior temporal sulcus, anterior part	51	−3	−21	BA21/22	5.52	35
R	Inferior temporal gyrus	57	−36	−18	BA21	5.24	18
R	Fusiform gyrus	45	−42	−21	BA21	4.42	
R	Middle temporal gyrus	54	−54	0	BA19/37	4.27	18
R	Caudate nucleus	9	18	0		7.31	54
L	Caudate nucleus	−9	9	6		5.99	151
L	Ventral anterior thalamus nucleus	−3	−9	0		5.81	

Peak voxel and cluster size that were significantly activated in the whole-brain random effects group analysis during the observation of social interaction displays versus non social interaction displays. t values reflect the statistical difference between the 2 conditions. The activations presented survived to correction for multiple comparisons (FDR) across the whole brain at *p<0.05*. Only activations in excess of 10 voxels are listed. L/R: left and right hemispheres.

Temporal activations were detected bilaterally in the posterior superior temporal sulcus (pSTS) and the right anterior superior temporal sulcus (aSTS). We also observed activations in the left TPJ. The right inferior temporal gyrus and right middle temporal gyrus also showed changes in activity. Frontal activations included the left dorsal part of the medial prefrontal cortex (MPFC) and the orbitofrontal cortices (OFC). Increased levels of activity were also observed in the fronto-parietal regions: activation occurred in the inferior frontal gyri (IFG) (*pars triangularis* and *pars opercularis*) and the premotor areas (PM), and the parietal activation included the right superior parietal gyrus (SPG) and the bilateral intraparietal sulci (IaPS). In the medial wall, activations were also observed bilaterally in the supplementary motor areas (SMA). More unexpectedly, activity was also elicited in the caudate nuclei within the basal ganglia.

## Discussion

The experimental procedure used in the present study made it possible to specifically isolate the social content carried by whole-body movements. Watching two agents engaged in a social interaction, as compared to observing non-social movements performed by the same agents, enhanced the recruitment of a region classically engaged in the processing of whole-body human motion (the pSTS), of structures belonging to the mentalizing network (the left TPJ, the right aSTS and the dorsal part of the MPFC), and of areas belonging to the action observation/execution matching network (the IFG, PM and IaPS bilaterally and the right SPG). Interestingly, we found the mentalizing network and the mirror network concomittantly activated while grasping the meaning of social intentions carried out by whole-body motions.

Despite a minimalistic presentation, the observers were able to determine whether or not the scene depicted a social interaction; perceiving social information such as emotion or intention is related directly to the movement kinematics [10], [40], [41]. Recently, the analysis of the kinematics of a gesture as simple as reaching towards an object and grasping it has shown differences in whether the gesture was associated with an intent to communicate or not [40]. In a previous study designed to categorize various types of human motion, non social human actions such as walking or climbing stairs were recognized more accurately than social actions such as greeting another person and dancing [4].

On the contrary, non-ambiguous stimuli were selected from the preliminary pre-fMRI study in the two conditions. The performance analysis confirmed that both were equally easy to categorize. The possibility can therefore be ruled out that the difficulty of the task may have been a confounder explaining some of the differences in brain activations. Lastly, greater arousal towards the social interaction condition, which presents with more interesting, salient and therefore endogeneously attracting features, might induce increased activity in regions involved in sustained attention expressed by increased saccades, such as the Frontal Eye Field (FEF) [42]. Indeed, eye movements reflected by FEF activity are made to orient our gaze and attention in order to provide salient cues to our on-going behavior [43]. A careful examination of the dorsolateral prefrontal activities indicated that these were outside the FEF. Nevertheless, we cannot exclude that some of the increased brain activations found when observing social interactions might reflect other additional attention processes.

### Greater recruitment of the pSTS classically processing whole-body point-light displays

One important point raised by our study is that observing whole-body human motion in a social context (emotional or not) activated the pSTS more than watching simple body movements. Besides its role in human motion processing [13], [15], [44], the pSTS is also known to be involved in cognitive processes such as detecting animacy and interactivity. It contributes to detecting whether or not a moving abstract shape is an animate being [45], and it participates in the spontaneous perception of animacy created by two interactive moving objects [46]. Both animacy (animated entities) and interactivity (interacting objects) are required for the attribution of agency, which has been defined as intentional contingencies between agents [47]. Detecting agency is crucial to distinguish the role of each of the interacting agents, and thus to grasp the significance of the social scene. It seems likely that the increased activity detected in the pSTS when subjects were viewing human motion depicting social interactions resulted from this process.

### Regions belonging to the mentalizing network contribute to attributing social meaning

Stronger activations were detected in the left TPJ, the right aSTS and the dorsal part of the MPFC when viewing the social scenes as compared to the non-social ones. These brain regions are generally attributed to mentalizing processes. Here we try to define more precisely their role in the understanding of a social interaction between two agents.

The TPJ region is thought to be involved in the computation of various spatial perspectives between the self and others, such as perspective taking [48], which makes it a good candidate brain region for mindreading. However, although some authors have stressed the role of the right TPJ in the attribution of mental states [49], we clearly observed the occurrence of a left hemispheric TPJ activation in the social condition. This is in line with the increased activation of the left TPJ detected by Ciaramidaro et al. [50] while subjects observed static images depicting “communicative intentions”, a condition which was fairly similar to our own social condition. The latter authors suggested that the right TPJ seems to be necessary for understanding “private intentions”, whereas the left TPJ may be specifically involved in understanding social intentions [50].

We also found strong bilateral activations in the dorsal MPFC, classicaly associated with tasks requiring thinking about oneself or others, and especially with judgments about people who are dissimilar to oneself [32]. Both studies using static cartoons describing communicative intentions have shown that the MPFC is involved in the processing of social interactions [50], [51], and video clips depicting social interactions activate the dorsal part of the MPFC [52]. Added to these previous findings, our study suggests that regardless of the amount of information provided by visual stimuli (realistic dynamic or static features, or minimalistic human shapes as in this study) the dorsal MPFC plays a major role in understanding social interactions.

### Activations within the mirror system are enhanced when watching social interactions

As compared to non social stimuli, social scenes elicited an increased level of activation in a fronto-parietal network including the IaPS, the PM areas and the IFG as part of the mirror system. We hypothesized the recruitment of the mirror system to be enhanced during the social scenes because they call for more complex action representations than meaningless movements. The build-up of action representations is a slow process that takes place during the entire childhood [53] and might be impaired in neurodevelopmental pathologies presenting with social deficits, such as autism spectrum disorders [54]. Likewise, the repertoire of social representations is fed by motor and social experience, raising it to a complex representational system in adults. It is therefore not surprising that the involvement of the mirror system translates the complexity of these representations.

Recently, activity within the rostral SMA (rSMA) has been associated with the action observation/execution matching network [55], [56]. The rSMA is not classically thought to be part of the mirroring system, but we also found it to be strongly co-activated with the fronto-parietal network, which supports the idea that this structure is involved in motor simulation. Observing grasping actions which intentions depend on the context [57] and observing actions with incongruent intentions elicit greater activations of the IFG [58]. The IaPS plays a pivotal role in intentional processes by translating encoded information into action [59]. Therefore, the mirroring system does not only provide an action recognition mechanism, but also constitutes a neural system for coding the intentional actions of others. The IaPS might also perform specific functions relating to non-verbal communication. Interestingly, the IaPS is selectively recruited by the processing of meaningful upper limb movements [60]. Therefore, the strong bilateral IaPS activation triggered when sujects are watching two characters engaged in a social interaction might also reflect a higher gesture processing, which contributes importantly to understanding social intentions.

### The motor simulation theory in social cognition

According to the motor simulation theory, people understand each other's actions by mapping the movements they see onto their own action representations [23], [25]–[28]. Our findings stress the role of motor simulation in understanding social intentions i.e. intentions directed towards a conspecific who acts back. Although it has been clearly established that understanding others' social intentions calls on brain regions which are partly associated with the mentalizing network, the contribution of motor resonance by means of the activation of the action observation/execution matching network (i.e. mirror system) is still a controversial issue. Some authors have claimed that the recognition of social intentions is an inferential or deductive process which activates regions belonging to the mentalizing network, which are located well outside the motor system [2], [29]–[30], [61]; whereas for others the ability to understand social intentions may be subtended by the mirror system [32], [62]–[66]. Recent experimental studies have pointed out that the mirror system may play a complementary role in the understanding of intentional actions [58], [67], [68] and during joint actions [69], [70]. A computational framework has been developed to explain how this system might provide the basis for reading the mental states of other people during action observation [71]. We provide here experimental evidence supporting the involvement of the mirror system in the understanding of social intentions between two interacting agents.

### The mirror and mentalizing networks concurrently active

While the mirror and mentalizing networks are rarely concurrently active [72], we found that, under specific conditions, both of them might be needed to catch the social intentions carried by whole-body motion. In the absence of biological motions the mirror system might not aid the mentalizing system in detecting intentionality [72]. While our experimental design enabled to subtract whole-body motion *per se* (SI condition versus NSI condition), we still found the mirror system activated, therefore underlining its contribution to social understanding. On the contrary, only the mentalizing network, but not the mirror system, was found active when participants were explicitly instructed to deliberate on the intentions of observed actors [58] or when they saw actions with unusual goal [29]. We demonstrated here that the mentalizing network and the mirror system might both be activated by high levels of goal i.e. social intentions. Co-activations of the mirror and mentalizing networks strongly support the hypothesis that they share a common functional core in social cognition. The precise interactions between these two brain networks and their development during childhood remain an open question which is of immense value for our understanding of social cognition.

## Supporting Information

Video S1 Example of social interaction under the social norm heading.
(AVI)

Video S2 Example of social interaction under the emotional situation heading.
(AVI)

Video S3 Example of social interaction under the game heading.
(AVI)

Video S4 Example of a display without social interaction.
(AVI)

Video S5 Example of a display without social interaction.
(AVI)

Video S6 Example of a display without social interaction.
(AVI)
